# Metastatic Squamous Cell Carcinoma of the Cervix Presenting With Haematemesis and Duodenal Obstruction: A Case Report and Literature Review

**DOI:** 10.1155/crgm/4772159

**Published:** 2026-05-05

**Authors:** D. L. Moodley, A. C. Van Wyk, M. Y. Sungay, A. A. Abdelsalem, M. S. Gabriel

**Affiliations:** ^1^ Division of Gastroenterology, Tygerberg Hospital, Stellenbosch University, Cape Town, South Africa, sun.ac.za; ^2^ Division of Anatomical Pathology, Tygerberg Hospital, Stellenbosch University, Cape Town, South Africa, sun.ac.za

**Keywords:** cancer, cervical, gastric, malignant, obstruction, outlet

## Abstract

**Background:**

Metastatic spread of cervical squamous cell carcinoma (SCC) to the duodenum is exceedingly rare, with fewer than 25 cases reported in the literature. Such metastases can present with gastrointestinal symptoms, including upper gastrointestinal bleeding or gastric outlet obstruction (GOO).

**Case Presentation:**

We report the case of a 58‐year‐old female with a history of Stage IIB cervical cancer in remission, who presented with haematemesis. Initially, this was ascribed to peptic ulcer disease but was later diagnosed as GOO due to a duodenal mass. Histology confirmed metastatic p16‐positive SCC, in keeping with metastatic cervical cancer. The patient’s malignant GOO was palliated successfully with the placement of a self‐expanding metal stent (SEMS). She died 1 month after discharge.

**Conclusion:**

This case highlights an uncommon metastatic pattern of cervical cancer, as well as the diagnostic challenge it can represent. In patients with prior malignancy, a high index of suspicion should be maintained when they present with GOO, regardless of the time interval. It also demonstrates the role of enteral stenting as an effective palliative approach in malignant GOO.

## 1. Introduction

In South Africa, the most commonly diagnosed nonskin cancers in women are breast, cervical and colorectal cancer [1]. In 2020, cervical cancer accounted for 10,702 new cases and 5870 deaths, with squamous cell carcinoma (SCC) accounting for the majority [2, 3]. Metastases to distant organs occur via haematogenous spread, predominantly to the lungs, liver and bones, lymphatic spread to pelvic and para‐aortic lymph nodes and direct invasion of adjacent organs [4]. Metastatic spread to the duodenum is rare [5]. The proposed mechanisms include direct invasion from metastatic retroperitoneal lymph nodes into the duodenum, as well as haematogenous dissemination resulting in mucosal implantation. To date, 22 cases have been reported in the literature (Table 1). A literature search was conducted using PubMed and Google Scholar from January 1980 to January 2025.

**TABLE 1:** Reported cases of duodenal metastases from cervical squamous cell carcinoma (SCC).

#	Reference	Age	Initial stage and prior treatment	Interval to metastasis	Symptoms	Treatment of metastasis	Outcome
1	Gurian L (1981)	64	Stage IIIB; none	Synchronous	Occult bleeding	None	DOD shortly after diagnosis (exact duration not specified)
2	Lee TH (2011)	50	Stage IIA; hysterectomy and chemotherapy	2 years	Abdominal pain	Chemotherapy	Not reported
3	Kanthan R (2011)	49	Stage IIA; chemoradiation	2 years	Massive upper gastrointestinal bleeding	None	DOD at 2 days
4	Raphael JC (2011)	57	Stage IV; chemoradiation	Synchronous	Recurrent vomiting and constipation	Nasojejunal feeding tube	Not reported
5	Kim SY (2012)	48	Stage IV; NA	3 months	Abdominal pain and vomiting	Uncovered SEMS	Not reported
6	Chawhan SM (2015)	52	NA; hysterectomy and radiation therapy	2 years	Abdominal pain, heartburn and nausea	None	Not reported
7	Lee JW (2015)	48	Stage IVB; chemotherapy	1 year	Abdominal pain	Second‐line chemotherapy	Not reported
8	Subramanian K (2016)	50	Stage IIA2; radiation therapy	2 years	Dyspepsia and abdominal pain	Chemotherapy	Alive, asymptomatic at follow‐up
9	Nag P (2016)	67	Stage IIB; chemoradiation	4 years	Abdominal pain and vomiting	Chemotherapy	DOD (exact duration not specified)
10	Qureshi T (2017)	54	NA; hysterectomy and chemoradiation	Not reported	Anorexia, early satiety and weight loss	None	Not reported
11	Shen CI (2017)	73	Stage IIB; chemoradiation	6 months	Abdominal pain and nausea	Uncovered SEMS	DOD at 1 month
12	Caparo AB (2018)	47	Stage IIB; radiotherapy, chemotherapy and brachytherapy	5 years	Abdominal pain, nausea and vomiting and weight loss	Palliative gastrojejunostomy	Not reported
13	Ash J (2021)	81	Stage IVA; radiation therapy	3 years	Abdominal pain	SEMS	DOD (exact duration not specified)
14	Lin YH (2022)	49	Stage IVB; hysterectomy and chemoradiation	6 years	Refractory nausea, vomiting, anorexia, abdominal pain and weight loss	Gastrojejunostomy with adjuvant chemotherapy	Disease‐free at 12 months
15	Marra E (2021)	38	NA; hysterectomy and chemoradiation	NA	Referred for duodenal stenosis	Uncovered SEMS	Not reported
16	Patel F (2021)	42	Stage IIIB; surgery and chemoradiation	2 years	Symptomatic anaemia and haematochezia	None	DOD at 6 weeks
17	Chen YH (2022)	66	Stage IB; hysterectomy and radiation therapy	9 years	Nausea and vomiting	Uncovered SEMS	Not reported
18	Narayan R (2022)	47	Stage IIIB; chemoradiation	7 months	Vomiting and constipation	None	Not reported
19	Green WC (2022)	59	NA	2 years	Abdominal pain	Chemotherapy	DOD at 6 months
20	Xiong SZ (2022)	67	NA; surgery and external radiation	10 years	Abdominal pain, nausea and vomiting	Targeted therapy and immunotherapy	Not reported
21	Thongpiya J (2024)	65	Stage IIIC; chemoradiation	4 years	Epigastric pain, nausea and vomiting	SEMS	Not reported
22	Al Ayoubi M (2025)	46	NA; surgery and chemoradiation	NA	Multiple episodes of haematemesis	None	DOD at 1 week
23	Moodley D (2025)– present case	58	Stage IIB; chemoradiation	5 years	Haematemesis, symptomatic anaemia and symptoms of gastric outlet obstruction	Uncovered SEMS	DOD at 1 month

Abbreviations: DOD = died of disease; NA = not available; SEMS = self‐expanding metal stent.

Despite its rarity, duodenal metastasis from cervical cancer may be underrecognised in clinical practice due to nonspecific gastrointestinal symptoms and often a prolonged latency period following initial treatment. This may result in diagnostic delay or misattribution of symptoms to more common benign conditions, particularly in patients thought to be in remission. There is therefore a need to increase clinical awareness of this entity, particularly among clinicians evaluating patients with a prior history of malignancy who present with features of GOO. This report aims to highlight this diagnostic challenge and contribute to the limited body of literature describing this unusual metastatic pattern. We present a case from South Africa, the 23^rd^ case.

## 2. Case History

A 58‐year‐old female presented to the emergency centre with signs and symptoms of an upper gastrointestinal tract (GIT) bleed in the form of haematemesis and symptomatic anaemia. She had been seen 6 weeks prior at a district hospital for similar symptoms, at which time an upper endoscopy was performed, confirming a prepyloric Forrest III ulcer. She was given *Helicobacter pylori* eradication therapy during her admission. She subsequently developed a deep vein thrombosis in her right leg whilst in the ward and was discharged on warfarin. However, the patient stopped her medication 3 days after discharge, making anticoagulation haemorrhage unlikely as the cause for her re‐presentation.

Her past medical history was significant for hypertension (on three agents) as well as previous cervical cancer Stage IIB. She had been diagnosed 5 years earlier and managed with chemoradiation after which she had remained in remission.

During her admission at our hospital, an oesophagogastroduodenoscopy (OGD) was attempted and aborted twice (due to the presence of a large amount of food in the stomach).

## 3. Differential Diagnosis, Investigations and Treatment

At this point, gastric outlet obstruction (GOO) was suspected, and the patient retrospectively reported additional symptoms of early satiety, postprandial fullness and weight loss.

### 3.1. OGD

**FIGURE 1:**
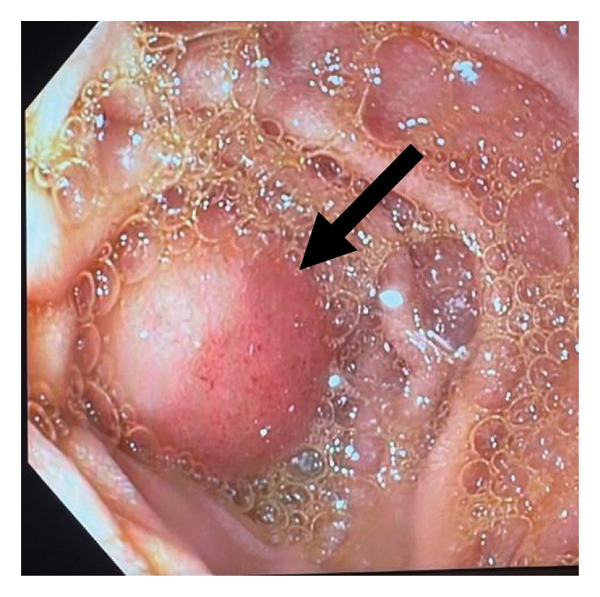
Endoscopic findings. Mass in the second part of the duodenum that was biopsied.

On the third OGD, she was noted to have oesophagitis, a small hiatus hernia, pangastritis and a mass in the second part of the duodenum causing luminal narrowing. This mass, not clearly separate from the ampulla of Vater (see Figure 1), was biopsied. It was possible to manoeuvre the scope through, suggesting a partial GOO.

### 3.2. Computed Tomography (CT) Imaging

**FIGURE 2:**
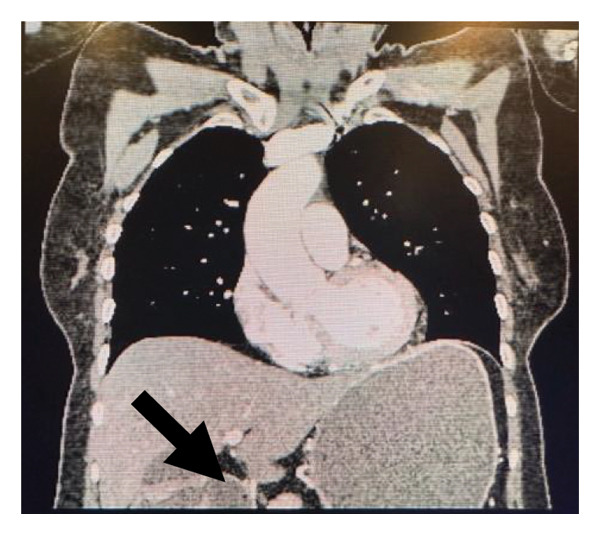
CT coronal view. Arrow showing the stenosis with upstream gastric dilatation.

A contrasted CT (chest and limited abdomen) confirmed a retroperitoneal, lobulated and heterogeneously enhancing soft‐tissue mass. This mass invaded the duodenum, displaced the pancreatic head and encased the distal abdominal aorta (with resultant dilatation of the proximal duodenum and stomach), see Figure 2. No distant metastases were seen on imaging. In addition, bilateral external iliac venous thromboses were noted.

### 3.3. Histology

**FIGURE 3:**
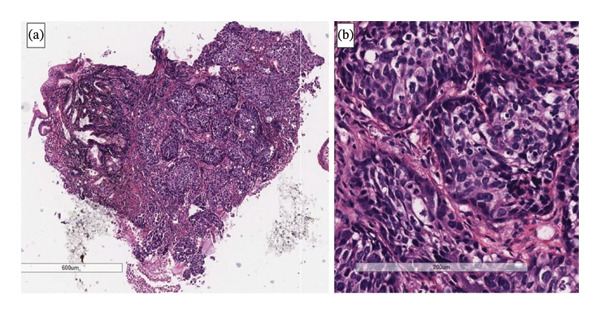
Histology. (a) Nests of tumour cells in the mucosa and submucosa mimic the nested pattern of a neuroendocrine tumour at low magnification. Haematoxylin and eosin, original magnification 4x. (b) At higher magnification, the tumour cell nuclei are more pleomorphic than typically seen in neuroendocrine tumours, and frequent apoptotic bodies are present. Keratinisation is not observed. Haematoxylin and eosin, original magnification 20x.

Histology showed nests of malignant epithelioid cells in the duodenal mucosa that stained positive for p63, p16 and CK5 (see Figure 3). Diffuse p16 overexpression in SCC on immunohistochemistry serves as a surrogate marker for high‐risk human papillomavirus (HPV)–associated malignancy. The high‐risk HPV oncoprotein E7 inactivates the retinoblastoma pathway, leading to upregulation of p16, which manifests as strong block‐type nuclear and cytoplasmic staining on immunohistochemistry. In the present case, diffuse p16 positivity in conjunction with p63 and CK5 expression supports metastatic HPV‐associated SCC. It strongly favours a primary cervical rather than a primary duodenal neoplasm [6].

### 3.4. Clinical Course

After discussion at the multidisciplinary meeting, it was decided to manage her palliatively. She developed worsening symptoms of GOO with minimal oral intake and intractable vomiting. An uncovered self‐expanding metal stent (SEMS) was deployed into the duodenum to relieve the obstruction.

### 3.5. Outcome and Follow‐Up

She did well postprocedure and was able to tolerate a full ward diet by the time she was discharged a week later.

The patient died at home 1 month later.

## 4. Discussion and Review of Literature

Tumours of the small bowel are unusual and account for only up to 5% of all GIT malignancies. Of these, metastatic lesions are more common than primary neoplasms. Cervical SCC typically spreads via direct extension, lymphatic spread or via haematogenous dissemination, most commonly to the lungs, liver and bone [3]. Spread to the duodenum is thought to occur through direct extension from involved para‐aortic lymph nodes or by haematogenous spread [7]. Metastatic cervical cancer carries a very poor prognosis [8]. Metastases to the duodenum remain infrequently reported, with only 22 cases published to date.

A review of reported cases (see Table 1) reveals several consistent patterns. The interval from primary diagnosis to duodenal involvement varies considerably, ranging from synchronous presentation to 10 years, although most occur within 2–5 years. In this case, metastasis occurred 5 years after the initial diagnosis of Stage IIB disease. This highlights the potential for late and unpredictable metastatic spread.

Detailed analysis of these reported cases demonstrates that the majority of patients present with nonspecific upper gastrointestinal symptoms. Abdominal pain, nausea and vomiting (often reflecting GOO) were the most common complaints, occurring in approximately 52% of patients. Gastrointestinal bleeding was less frequent, reported in a minority of patients, while a small number presented with atypical or mixed symptoms such as anaemia or weight loss. This variability in presentation likely contributes to diagnostic delay, particularly in patients with a remote history of malignancy. Imaging and endoscopy are pivotal in identifying lesions, but definitive diagnosis hinges on histology and immunohistochemical staining.

Our patient presented with a prepyloric ulcer 6 weeks prior to current admission; this could be indicative of early obstruction, metastatic disease in evolution or could have been an incidental finding. When she developed haematemesis, it was most likely due to tumour‐related bleeding, as she was not on anticoagulation at the time and had no ulcer on repeat upper endoscopy.

Therapeutic modalities for malignant GOO have evolved over time: earlier reports favoured conservative or surgical strategies, whereas more recent cases increasingly use minimally invasive interventions. The therapeutic options include surgical gastrojejunostomy (either open or laparoscopic), endoscopic ultrasound (EUS)–guided gastrojejunostomy and enteral stenting using SEMS [9]. These modalities differ in terms of technical success, clinical durability and suitability depending on patient factors and life expectancy. A large meta‐analysis of patients with malignant GOO from multiple types of cancer, however, demonstrated similar short‐term morbidity and mortality when comparing the different interventions [10].

Surgical gastrojejunostomy offers durable long‐term patency and lower reintervention rates, but is associated with higher upfront morbidity and longer recovery time, making it less suitable for patients with advanced disease and limited survival. EUS‐guided gastrojejunostomy has emerged as an effective minimally invasive alternative, demonstrating high technical and clinical success rates and lower reintervention rates than SEMS, although its availability is limited to specialised centres and requires advanced endoscopic expertise. Enteral stenting with SEMS is widely used due to its high technical success, rapid symptom relief, and minimally invasive nature. Uncovered stents are preferred in these cases, as they are less prone to migration and allow for biliary flow, although they can be complicated by tumour ingrowth and eventual obstruction. Recent evidence, including a contemporary systematic review [11], suggests that while EUS‐guided gastrojejunostomy may offer superior long‐term patency, SEMS remains the preferred option in patients with limited life expectancy due to its lower procedural burden and faster recovery. SEMS is therefore an appropriate first‐line palliative intervention in selected patients. It was the most frequently employed modality among reviewed cases due to its minimally invasive nature and effectiveness in alleviating GOO.

Our patient was assessed as a poor surgical candidate due to advanced disease, poor functional status and guarded prognosis. In addition, SEMS is more readily available than EUS‐guided gastrojejunostomy at our institution. Enteral stenting was therefore deemed the best treatment option, and a duodenal SEMS was successfully placed.

Despite therapeutic advances, survival outcomes remain poor overall, with the majority of patients dying within weeks to months of diagnosis. Only a small number of cases report longer survival, typically patients who underwent combined multimodal therapy. Our case demonstrates the aggressive nature of metastatic cervical cancer. Our patient underwent successful SEMS placement but died 1 month later, mirroring the poor outcome seen in many of these cases. Only a few case reports describe prolonged survival following combined surgical and chemotherapeutic management.

## 5. Conclusion

This case contributes to the limited body of literature describing duodenal metastases from cervical cancer and highlights an uncommon, yet clinically significant metastatic pattern. In patients unfit for surgery, enteral stenting represents a safe, effective and minimally invasive option for palliation of malignant GOO.

**Nomenclature**
GOO: Gastric outlet obstruction
SCC: Squamous cell carcinoma
GIT: Gastrointestinal tract
SEMS: Self‐expanding metal stent
OGD: Oesophagogastroduodenoscopy
CT: Computed tomography
HPV: Human papillomavirus
EUS: Endoscopic ultrasound

## Author Contributions

A. A. Abdelsalem, A. C. Van Wyk and D. L. Moodley contributed to the management of this patient and the concept of this case report. D. L. Moodley and A. C. Van Wyk performed the literature review and drafting of the case report. A. C. Van Wyk, D. L. Moodley, M. S. Gabriel and M. Y. Sungay assisted with the editing and revision process.

## Funding

No funding was required.

## Disclosure

All authors have approved the final version.

## Consent

A waiver of informed consent has been approved by the Stellenbosch Health Research and Ethics Council (reference C25/07/027).

## Conflicts of Interest

The authors declare no conflicts of interest.
